# Impact of high-resolution 3D-mapping with micro-electrodes on catheter ablation of Wolff-Parkinson-White syndrome

**DOI:** 10.1016/j.ijcha.2024.101435

**Published:** 2024-06-05

**Authors:** Johannes Steinfurt, Alexander Gressler, Judith Stuplich, Eike Jordan, Markus Jäckel, Diona Gjermeni, Martin Eichenlaub, Marius Bohnen, Armin Luik, Amir Jadidi, Thomas S. Faber, Brigitte Stiller, Dirk Westermann, Thomas Arentz, Heiko Lehrmann, Denis Fedorov

**Affiliations:** a Department of Cardiology and Angiology, University Heart Center Freiburg – Bad Krozingen, Faculty of Medicine, University of Freiburg, Freiburg, Germany; b Medizinische Klinik IV at Städtisches Klinikum Karlsruhe, Academic Teaching Hospital of the University of Freiburg, Germany; c Department of Pediatric Cardiology, University Heart Center Freiburg – Bad Krozingen, Faculty of Medicine, University of Freiburg, Freiburg, Germany

## Abstract

**Background:**

It is currently unknown whether *high-resolution* 3D-mapping and micro-electrodes add meaningful benefits in catheter ablation of Wolff-Parkinson-White (WPW) syndrome and challenging, e.g. *para*-Hisian accessory pathways (APs).

**Objectives:**

To compare the mapping resolution, acute success and complication rates in patients with WPW syndrome undergoing a first-time catheter ablation using only a contact force-sensing ablation catheter for mapping or a multi-electrode high-resolution mapping catheter.

**Methods:**

Fifty consecutive 3D-mapping procedures for WPW syndrome using a 3.5-mm ablation catheter (n = 27) or a multi-electrode high-resolution catheter (n = 23) were retrospectively analyzed regarding mapping resolution defined as first 5/10 msec isochronal activation area, number of RF applications to achieve AP block, occurence of AP automaticity during RF delivery, and acute success and complication rates.

**Results:**

Catheter ablation was successful in 48/50 patients with a median of 1 (IQR 1–2) RF applications. Compared to ablation catheter mapping, high-resolution mapping showed a significantly smaller isochronal activation area in the first 5/10 msec (1.25 ± 0.29 vs 0.15 ± 0.03 cm^2^; *P* < 0.001 and 3.41 ± 0.58 vs 0.55 ± 0.12 cm^2^; *P* < 0.0001) and significantly higher incidence of AP automaticity during RF delivery (0 vs 22 %; *P* < 0.05). In *para*-Hisian APs, micro-electrodes recorded distinct His electrograms and AP potentials without fusion and without AP bumping permitting safe and effective *para*-Hisian AP ablation.

**Conclusions:**

High-resolution mapping increases the mapping accuracy of the AP and its insertion site leading to a significantly higher incidence of AP automaticity during RF delivery. Micro-electrodes provide clinically relevant advantages in *para*-hisian AP mapping improving efficacy and safety of *para*-Hisian AP ablation.

## 1 Background

Three-dimensional electro-anatomical mapping is recommended for challenging accessory pathways (APs) [1]. In this context it is unknown whether high-resolution 3D-mapping and the use of micro-electrodes provide additional benefits. Due to the susceptibility to record fused electrograms (EGMs), ablation catheters with large 3.5-mm tip electrode appear to be suboptimal for AP mapping. In contrast, high-resolution 3D-mapping with closely-spaced micro-electrodes can record tiny AP potentials without atrial or ventricular electrogram fusion [2] which may be beneficial for mapping of challenging, particularly *para*-Hisian APs. The aim of this study therefore was to compare the mapping resolution, acute success and complication rates in Wolff-Parkinson-White (WPW) syndrome patients undergoing 3D-mapping and catheter ablation of atrioventricular and *para*-Hisian APs using only a contact force (CF)-sensing ablation catheter for mapping or a dedicated multi-electrode high-resolution mapping catheter.

## 2 Methods

### 2.1 Study population

We retrospectively analyzed 50 consecutive patients undergoing 3D-mapping and first-time catheter ablation for WPW syndrome by four experienced electrophysiologists at our institution from July 2022 – November 2023. Point-by-point mapping was mainly done at the Bad Krozingen site and high-resolution 3D-mapping was mainly done at the Freiburg site. All patients provided written informed consent for mapping and ablation and anonymized retrospective data collection was approved by the local institutional review board of the University of Freiburg, Germany. Indications for EP study and catheter ablation were based on the current ESC guidelines [3].

### 2.2 EP study

AADs were discontinued for at least 5 half-lives prior to the procedure. Conscious sedation with midazolam, fentanyl and propofol was used in the majority of patients. A 6F or 7F deca- or duodecapolar catheter (Inquiry, Abbott or CristaCath, Biosense Webster) was advanced in the coronary sinus (CS) and up to three 4F quadripolar catheters were positioned at the RV apex, His bundle and right atrial appendage. In case of inducible SVT, standard pacing maneuvers were carried out to verify AP participation in the SVT circuit. In patients with left-sided pathways, left atrial access was obtained via a PFO or transseptal puncture guided by fluoroscopy, transesophageal echocardiography, or intracardiac echocardiography (AcuNav, Siemens Medical Solutions). A Heparin bolus was given after groin puncture prior to left atrial access followed by continuous Heparin administration to maintain an ACT > 300 sec.

### 2.3 Mapping

Electro-anatomical maps were acquired using the CARTO3 system (Biosense Webster) during mainly paced rhythms ensuring conduction over the AP in order to measure the area activated in the first 5 or 10 msec. Point-by-point mapping was done with a ThermoCool SmartTouch with 3.5-mm tip electrode (n = 27). Multi-electrode high resolution 3D-mapping was performed with a Penta-/Octaray (n = 11/12) using closely spaced bipoles with 2-mm interelectrode spacing (1-mm ring- or 0.5-mm micro-electrodes with 2-6-2- or 2-2-2-2-2-mm edge-to-edge spacing). Adequate tissue contact was achieved by a CF > 5 g or by observing a physical deformation of the catheter splines in the mapping system when pressed against the tissue wall. A steerable 8.5F Agilis NxT (Abbott) or Vizigo (Biosense Webster) sheath was used to increase stability, e.g. in *para*-Hisian APs. In the high-resolution cohort points were continuously acquired with strict filter settings (intracardiac CS/QRS reference with pattern matching ≥ 90/98 %, cycle length/local activation time stability: ± 2 ms, position stability: ± 2 mm, tissue proximity indicator: on). Uni- and bipolar EGMs were filtered at 2–240 and 16–500 Hz, respectively. EGMs were automatically annotated to the maximal unipolar dV/dt (wavefront algorithm) and AP potentials and His signals were annotated manually. The fill color threshold and internal point filter were kept at a minimum of 5 mm to avoid interpolation and cavity far-field potentials. Open window mapping (OWM) [4] and the extended-early-meets-late feature were used to highlight the electrical annulus leaving a visible gap only in presence of AP potentials [2]. Fluoroscopy use was minimized following the ALARA principle [5].

### 2.4 Catheter ablation

The AP potential or the earliest 5 or 10 msec isochronal activation area, i.e. the AP body or its insertion site, served as ablation targets [6]. In all patients catheter ablation was performed via a transfemoral venous approach with power-controlled radiofrequency (RF) energy at 25–50 W using an irrigated CF-sensing Thermocool SmartTouch or Thermocool SmartTouch Surround Flow (Biosense Webster). If Adenosine testing had been negative (no recovery of ante- nor retrograde AP conduction), there was no systematic waiting period.

### 2.5 Statistical analysis

Statistical analyses were performed using GraphPad Prism, version 5.01. Categorical variables were reported as absolute and percentage values. Continuous data were presented as mean ± SD or mean ± SEM for normally distributed data and median (IQR) for nonnormally distributed data. Comparison of variables were analyzed by Student’s t, χ2, Fisher’s exact or Mann-Whitney test.

## 3 Results

Fifty mostly male patients aged 42 ± 17 years with a single AP were included in the analysis (Table 1). Most APs were manifest and located at the anterior to lateral mitral anulus followed by postero-septal and *para*-Hisian locations. Catheter ablation was successful in 48 of 50 patients (one failure in each group) with a median of 1 (IQR 1–2) RF applications (Table 2). There was no instance of AV block, two right bundle branch blocks (RBBB) associated with *para*-Hisian AP ablation (one in each group), and three minor groin complications. The mean fluoroscopy time and dose area product were lower in the ablation catheter group due to a refined ultra low-dose protocol and 30 % right posteroseptal APs with fluoroless approach. The mean procedure time was 120 ± 7 min with non-significant shorter procedure times associated with ablation (Abl) catheter mapping. There was no significant difference in the rate of AP potential detection (52 vs 52 %; *P* = 1) or AP elimination with the first RF delivery (52 vs 65 %; *P* = 0.76). However, high-resolution mapping showed a significantly smaller isochronal area activated in the first 5/10 msec (1.25 ± 0.29 vs 0.15 ± 0.03 cm^2^; *P* < 0.001 and 3.41 ± 0.58 vs 0.55 ± 0.12 cm^2^; *P* < 0.0001) and a significantly higher incidence of AP automaticity during RF delivery (0 vs 22 %; *P* < 0.05) (Table 2).

**Table 1:** Baseline patient and accessory pathway characteristics of n = 50 WPW syndrome patients undergoing 3D-mapping with only a contact force-sensing ablation catheter (Abl) or a multi-electrode high-resolution catheter (ME) for mapping.

**Parameter**	**All patients (n = 50)**	**Abl mapping** **(n = 27)**	**ME mapping** **(n = 23)**	*P* value*
**Patient characteristics**				
Age (y)	42 ± 17 (26–56)	41 ± 14 (30–54)	43 ± 20 (22–60)	0.65
Male sex	36 (72)	18 (66)	18 (78)	0.55
BMI (kg/m^2^)	26.7 ± 0.7	26.5 ± 0.8	26.9 ± 1.1	0.78
**Accessory pathway characteristics**				
Manifest	33 (66)	15 (55)	18 (78)	0.14
Decremental	1 (2)	0 (0)	1 (4)	0.45
Left anterior	5 (10)	4 (15)	1 (4)	0.38
Left antero-lateral	6 (12)	1 (4)	5 (22)	0.08
Left lateral	10 (20)	5 (19)	5 (22)	0.72
Left postero-lateral	4 (8)	1 (4)	3 (13)	0.32
Left posterior	2 (4)	1 (4)	1 (4)	1.0
Left postero-septal	3 (6)	2 (7)	1 (4)	1.0
Right postero-septal	9 (18)	8 (30)	1 (4)	0.03
Right-sided	4 (8)	2 (7)	2 (9)	1.0
Para-Hisian	7 (14)	3 (11)	4 (17)	0.69

Values are n (%), n, mean ± SD, mean ± SE, or median (IQR).

*P values by Student’s *t* test, χ2, Fisher’s exact or Mann-Whitney test.

P values < 0.05 were considered significant.

**Table 2:** Comparison of procedural, mapping and ablation data between n = 50 WPW syndrome patients undergoing 3D-mapping with only a contact force-sensing ablation catheter (Abl) or a multi-electrode high-resolution catheter (ME) for mapping.

**Parameter**	**All patients (n = 50)**	**Abl mapping** **(n = 27)**	**ME mapping** **(n = 23)**	*P* value*
**Procedural data**				
Acute success	48 (96)	26 (96)	22 (96)	1.0
Complications	5 (10)	2 (7,4)	3 (13,0)	0.64
Adenosine testing	38 (76)	18 (67)	20 (87)	0.11
Adenosine dose, mg	18.8 ± 0.5	17.9 ± 0.9	19.5 ± 0.7	0.18
Fluoroscopy time, min	5.3 ± 0.8	2.7 ± 0.7	8.2 ± 1.3	<0.001
Dose Area Product, µGy x m^2^	95.6 ± 16.9	34.1 ± 9.8	166.8 ± 28.7	<0.0001
Procedure time, min	120 ± 7	111 ± 10	138 ± 8	0.08
**Mapping and ablation data**				
AP potential	25 (50)	13 (52)	12 (52)	1.0
Isochronal area (initial 5-ms) , cm^2^	0.70 ± 0.17	1.25 ± 0.29	0.15 ± 0.03	<0.001
Isochronal area (initial 10-ms) , cm^2^	1.94 ± 0.37	3.41 ± 0.58	0.55 ± 0.12	<0.0001
RF deliveries to AP block	1 (1–2)	1 (1–2)	1 (1–2)	0.71
AP elimination with 1st RF	28 (61)	15 (52)	13 (65)	0.76
AP automaticity during RF	5 (10)	0 (0)	5 (22)	<0.05

Values are n (%), n, mean ± SE, or median (IQR).

*P values by Student’s *t* test, χ2, Fisher’s exact or Mann-Whitney test.

P values < 0.05 were considered significant.

## 4 Discussion

The present study sought to assess the role of high-resolution 3D mapping vs point-by-point ablation catheter mapping in WPW patients undergoing catheter ablation, with particular focus on the impact of micro-electrodes on *para*-Hisian AP mapping.

**Fig. 1:**
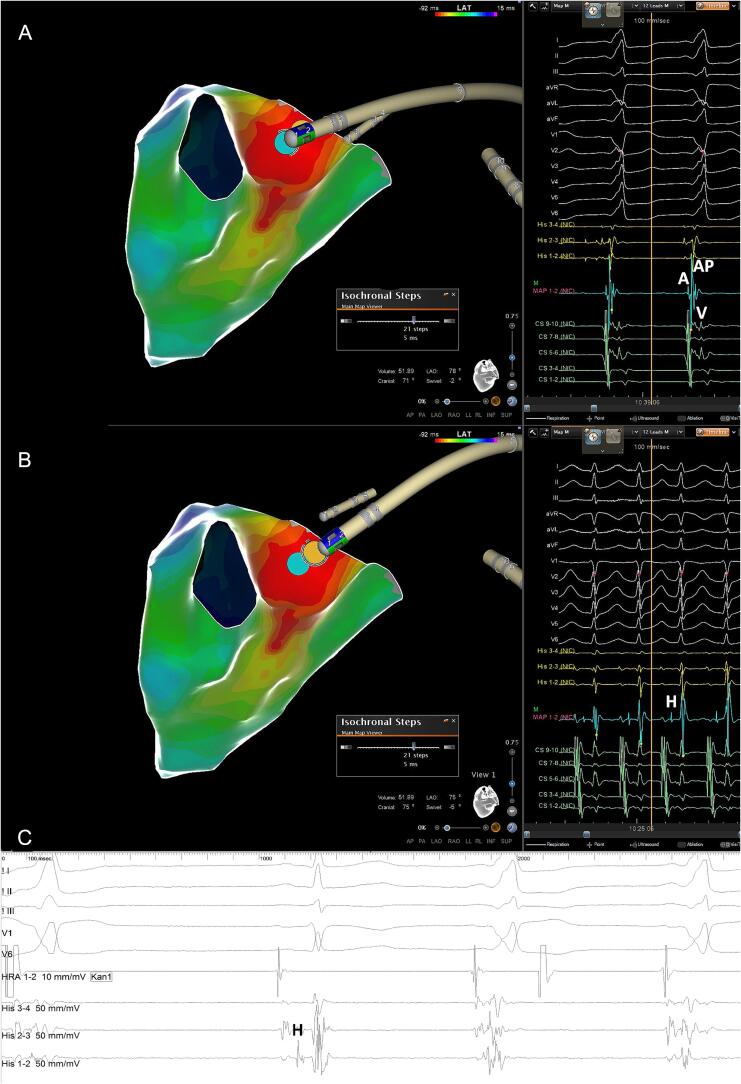
Point-by-point mapping of *para*-Hisian AP with (−) delta-wave in V1. A: Point-by-point mapping with the ablation catheter revealed a broad activation within the first 5 msec directed towards the septum leading to a negative delta-wave in V1. The AP potential (blue tag) is fused with the atrial and ventricular EGMs and a concealed His EGM cannot be ruled out. Note perpendicular catheter orientation with the femoral approach which led to persistent AP bumping during ablation catheter mapping when the patient took a deep breath. Catheter ablation at the bumping site was ultimately successful confirmed by absence of AP recovery with 24 mg Adenosine and no recurrence during follow-up. B: Distal His bundle localization (yellow tag) at the AP potential site (blue tag) during orthodromic reciprocating tachycardia. C: Concealed His EGM on quadripolar catheter due to fused atrial and ventricular EGMs during pre-excitation.

**Fig. 2:**
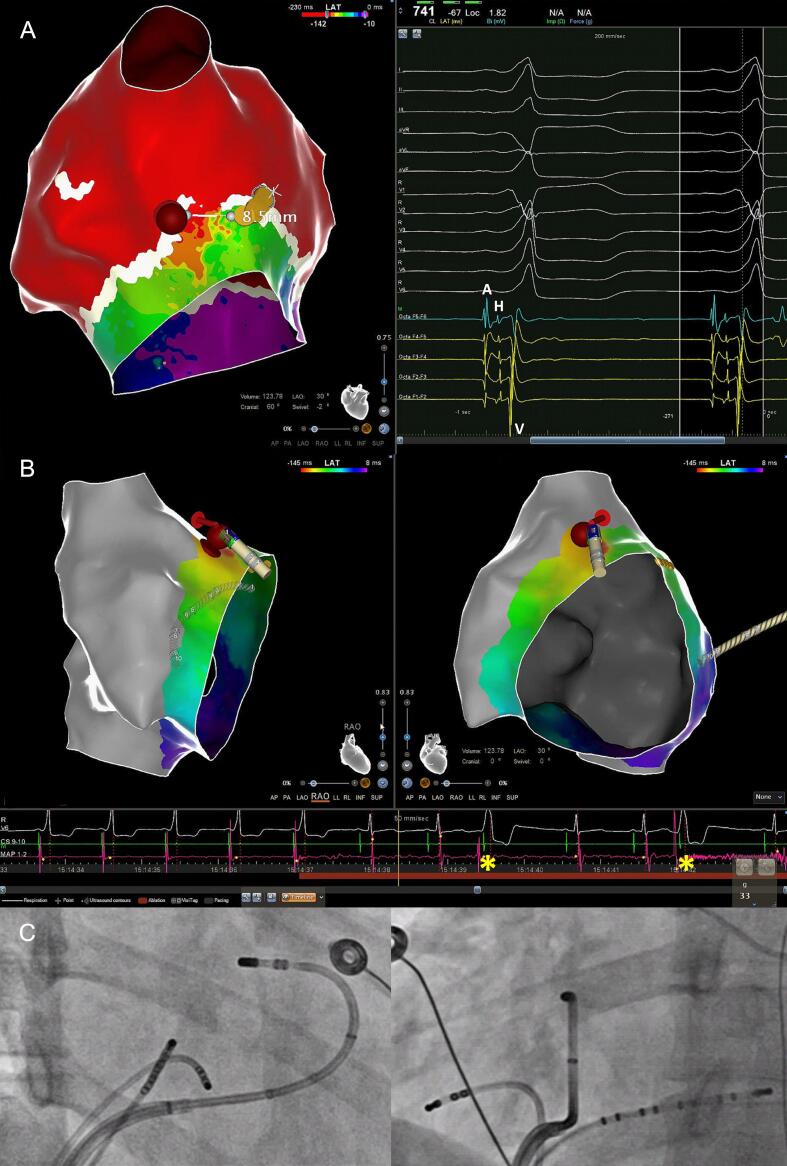
High-resolution mapping of *para*-Hisian AP with (+) delta-wave in V1. A: High-resolution OWM revealed the earliest activation with distinct AP potential *lateral* to the RV summit with an oblique course towards the free wall leading to a positive delta-wave in V1 (Video 1). Despite a distance of less than 5 mm between the distal His and the distal AP potential, *antegrade* His bundle activation (yellow tags) was clearly visible during pre-excitation along the adjacent Octaray micro-electrodes with close 2-mm spacing (Suppl Fig. 1). B: The first RF delivery targeting the proximal AP potential (red tag) led to instant loss of pre-excitation along with bi-directional AP automaticity. Note two beats with retrograde VA conduction (asterisks) and absence of any atrial electrogram on the ablation catheter. C: Corresponding fluoroscopic images in RAO and LAO demonstrating a stable catheter position (33 g mean CF) with the femoral approach.

**Supplementary figure 1:**
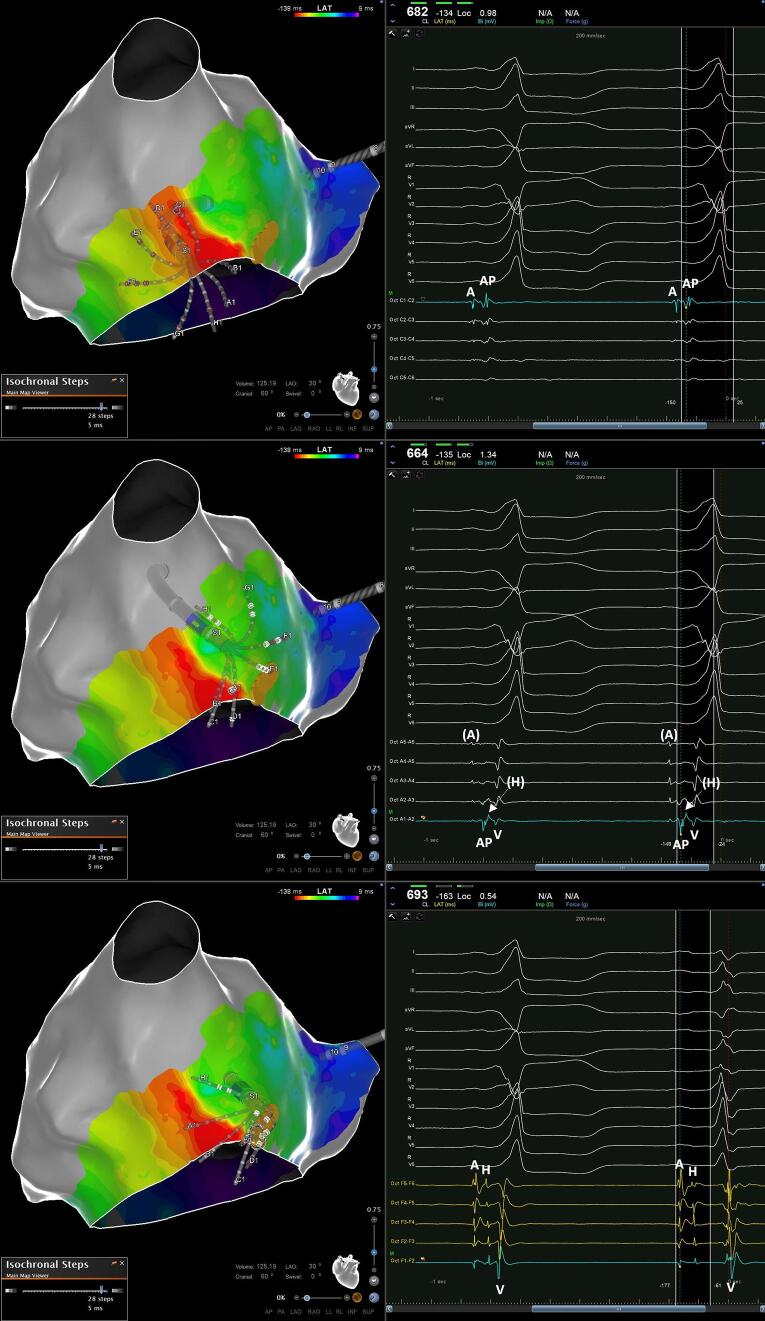
A and B: Distinct proximal/distal para-Hisian AP potentials without atrial/ventricular EGM fusion on distal C/A spline micro-electrodes with close 2-mm spacing and far-field His signal (arrow). C: Antegrade His bundle activation along F spline micro-electrodes with close 2-mm spacing during pre-excitation and RBBB. The first QRS complexes are aligned to enable EGM timing comparison.

Across all AP locations there was no significant difference between both mapping strategies regarding detection rates of AP potentials and AP elimination with the first RF delivery, stressing again the importance of a countercurrent paced wavefront to expose the AP potential as reported by Jackman [7]. Compared to Abl mapping, *high-resolution* mapping with closely spaced bipoles increases the mapping accuracy of the AP and its insertion site significantly which may be particularly relevant for *para*-Hisian APs where the His bundle is usually concealed by overlapping atrial and ventricular EGMs [22]. Indeed, various strategies to avoid AV block in these challenging pathways have been reported, e.g. by identifying the His bundle with ablation catheter-embedded micro-electrodes [8] and low-output near-field His capture [9], or by using distant ablation targets in the ventricle and aortic cusps [10], [11], [12]. Nevertheless, ablation catheters appear to have significant limitations for *para*-Hisian AP mapping: First, the relatively stiff 8F ablation catheter is 9x bigger than an AP (mean diameter 300 µm) [8], [13] and is oriented perpendicular to the tissue plane during mapping thereby increasing the risk of bumping which occurred in 3/7 of our patients (Fig. 1). Indeed, Berte et al also report *para*-Hisian AP bumping with the Abl in the same patient in three consecutive procedures [8] and AP bumping occurred in up to 20 % of anteroseptal APs in a large Chinese series with a 30 % recurrence rate after ablation at the bumping site [14]. Second, the large 3.5-mm tip electrode (surface area 27 mm^2^) is prone to record far-field signals and fused EGMs making a clear discrimination of atrial, ventricular, His and *para*-Hisian AP potentials challenging (Fig. 1 [10]). In contrast, the soft and flexible 2F Octaray splines and closely spaced micro-electrodes (surface area 0.9 mm^2^) are oriented parallel to the tissue plane which minimizes the risk of AP bumping while maximizing the electro-anatomical resolution [15], [16], [17]. The bipolar EGM resolution is further increased by an internal unipolar reference electrode that enhances the near- while reducing the impact of cavity far-field signals [2]. Indeed, this higher electro-anatomical resolution of the Octaray allowed to record *distinct antegrade His EGMs* without fusion despite pre-excitation (Fig. 2) and identified the exact *para*-Hisian AP localization and AP insertion site as evidenced by AP block in < 1 sec. of RF delivery (Fig. 2/3). Compared to the Pentaray catheter with closely spaced bipoles the Octaray with close 2-mm spacing allowed to determine the direction of impulse propagation with one catheter position (Suppl Fig. 1/3) which was particularly useful in *para*-Hisian APs to confirm the origin of EGMs as atrial, His and ventricular. While Abl mapping caused *para*-Hisian AP bumping in a signifcant proportion of our patients requiring a second procedure in 2/3, the soft and flexible 2F Octaray splines recorded > 20.000/40.000 points, respectively without AP bumping (Videos 1/2) permitting safe and effective *para*-Hisian AP ablation. Mapping the right bundle branch with the Octaray as previously shown by us [2] may also prevent RBBB in *para*-Hisian AP ablation.

**Fig. 3:**
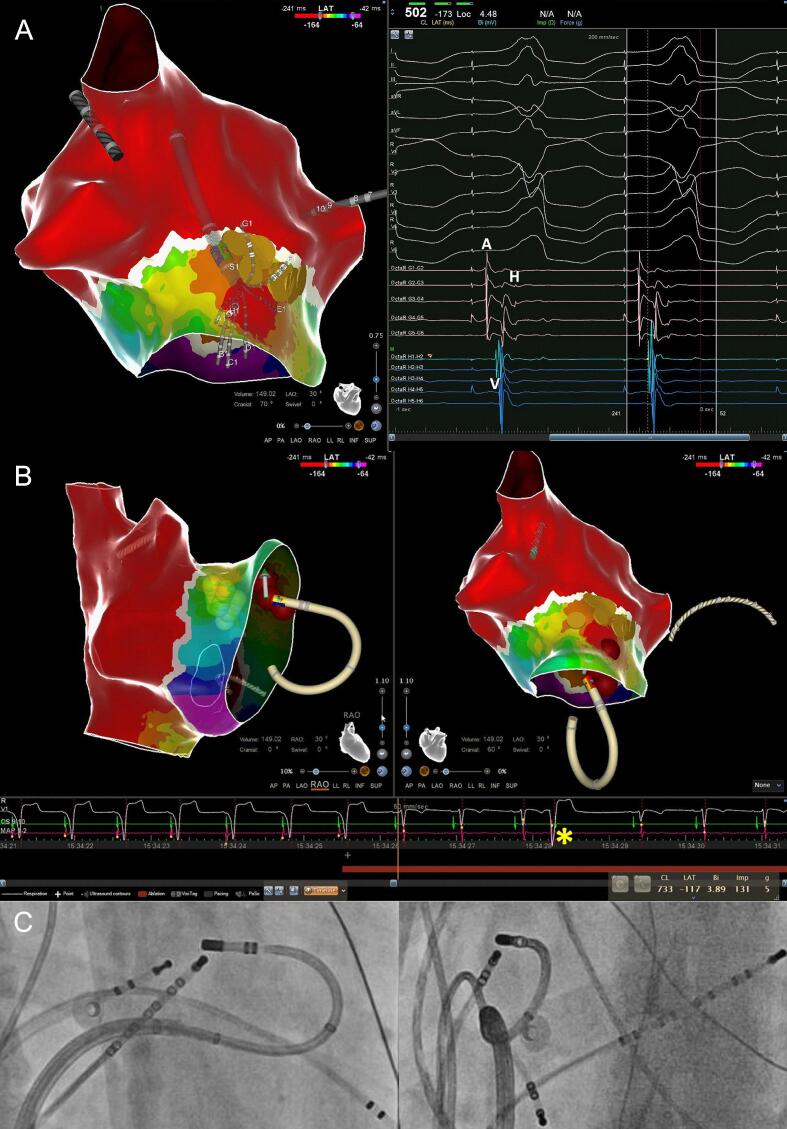
High-resolution mapping of *para*-Hisian AP with (−) delta-wave in V1 A: High-resolution OWM during atrial pacing revealed the earliest activation *septal* to the RV summit directed towards the septum leading to a negative delta-wave in V1 (Video 2). The distal His (yellow tags) was located during narrow QRS complex paced beats and was also seen on the G spline micro-electrodes with close 2-mm spacing during paced pre-excitation (A-V-H sequence with identical stim-His interval) (Suppl Fig. 2). B: The second RF delivery (with sufficient CF) targeting the ventricular pathway insertion with a possible AP potential (H1-H2 bipole) led to instant loss of pre-excitation and bi-directional AP automaticity. Note one beat with retrograde VA conduction (asterisk) and absence of any atrial electrogram on the ablation catheter. C: Corresponding fluoroscopic images in RAO and LAO demonstrating sufficient stability (5 g mean CF) with the femoral approach.

**Supplementary figure 3:**
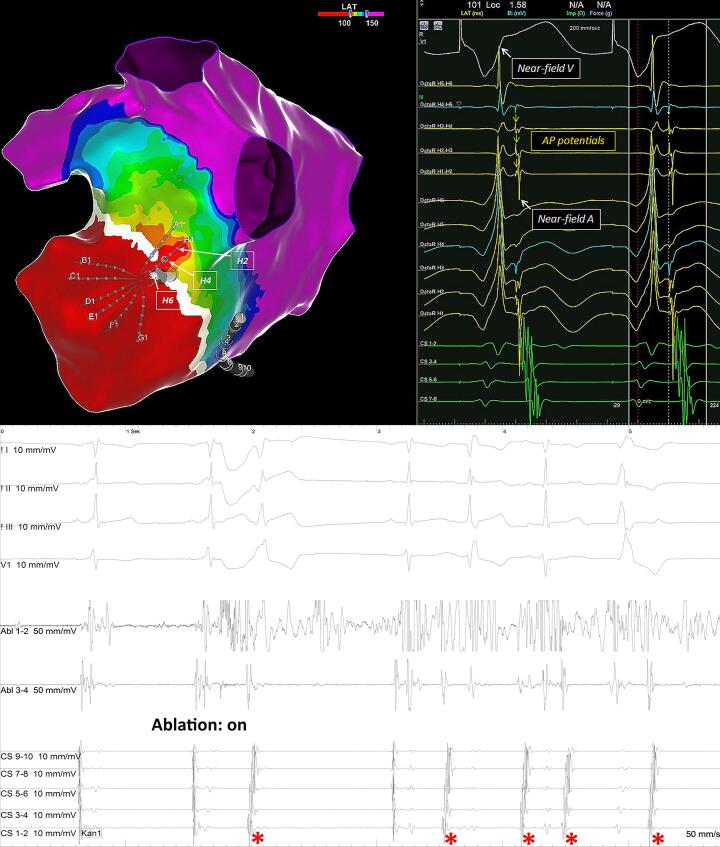
A: High-resolution open window mapping during RV pacing with sharp, isolated AP potentials at the lateral mitral anulus along the adjacent H spline micro-electrodes with close 2-mm spacing. B: The first RF delivery targeting the earliest, isolated AP potential (unipolar OctaR H4) led to instant loss of pre-excitation along with instant AP automaticity (asterisks) with eccentric CS activation similar to retrograde AP conduction during RV pacing.

AP automaticity is a relatively rare phenomenon that has been reported to occur only *after* RF delivery in atrioventricular APs [18], [19], [20], [21]. Another result of this study is the novel finding that high-resolution mapping is associated with AP automaticity *during* RF delivery in a significant proportion of atrioventricular APs similar to junctional and Mahaim automaticity in slow pathway and atriofascicular fiber ablation. AP automaticity was observed only with *high-resolution* mapping in 33 % of the Octaray procedures reflecting its high mapping resolution resulting in RF delivery directly to the AP (Fig. 1/2 and Suppl Fig. 3). We are aware of two experienced EP colleagues from Argentina and Germany, Dres. Alberto Alfie and Patrick Müller, who have made similar observations that corroborate our findings. Similar to slow pathway and Mahaim fiber ablation the implication may be that one should continue to deliver RF energy whenever AP automaticity occurs. Future studies are needed to assess the true prevalence of AP automaticity with the use of high-resolution mapping and whether occurrence and complete elimination of AP automaticity predicts long-term success in catheter ablation of WPW syndrome.

In summary, high-resolution mapping with micro-electrodes provides quantitative and qualitative advantages in AP mapping. In our experience there is an indication for the use of *high-resolution* mapping with micro-electrodes in high-risk APs located near the His bundle where atraumatic mapping and increased mapping resolution are critically important.

### 4.1 Study limitations

The limitations relate to the relatively small sample size and the non-randomized, retrospective nature of this study where point-by-point and multi-electrode mapping were mainly performed by different operators. In addition, AP potentials were not systematically validated by pacing maneuvers.

## 5 Conclusions

Multi-electrode catheters with closely spaced bipoles increase the mapping accuracy of the AP and its insertion site leading to a higher incidence of AP automaticity during RF delivery in catheter ablation of WPW syndrome. In *para*-Hisian APs, micro-electrodes provide clinically relevant advantages by recording distinct AP potentials and distinct His EGMs without fusion and without persistent AP bumping permitting safe and effective *para*-Hisian AP ablation.

## CRediT authorship contribution statement

**Johannes Steinfurt:** Writing – review & editing, Writing – original draft, Visualization, Validation, Project administration, Methodology, Investigation, Formal analysis, Data curation, Conceptualization. **Alexander Gressler:** Writing – review & editing, Investigation. **Judith Stuplich:** Writing – review & editing. **Eike Jordan:** Writing – review & editing. **Markus Jäckel:** Writing – review & editing. **Diona Gjermeni:** Writing – review & editing. **Martin Eichenlaub:** Writing – review & editing. **Marius Bohnen:** Writing – review & editing. **Armin Luik:** Writing – review & editing. **Amir Jadidi:** Writing – review & editing, Investigation. **Thomas S. Faber:** Writing – review & editing. **Brigitte Stiller:** Writing – review & editing. **Dirk Westermann:** Writing – review & editing. **Thomas Arentz:** Writing – review & editing, Conceptualization. **Heiko Lehrmann:** Writing – review & editing, Investigation, Conceptualization. **Denis Fedorov:** Writing – review & editing, Formal analysis, Data curation.

## Declaration of competing interest

The authors declare that they have no known competing financial interests or personal relationships that could have appeared to influence the work reported in this paper.

## Figures and Tables

**Supplementary figure 2:**
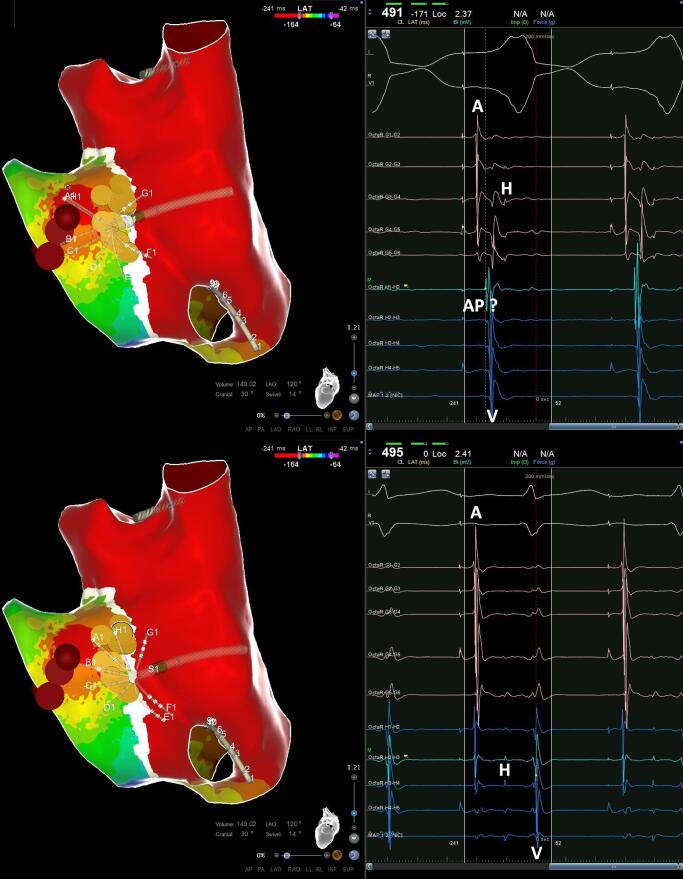
A: Earliest ventricular activation without atrial/ventricular EGM fusion with a possible para-Hisian AP potential on the distal H spline micro-electrodes with close 2-mm spacing. A and B: distal His localization with identical stim to His interval on G/H spline micro-electrodes during pre-excitation and narrow QRS. The first QRS complexes are aligned to enable EGM timing comparison.
